# AFFORDABLE, ACCESSIBLE HEARING CARE AMONG INDIVIDUALS WITH COGNITIVE IMPAIRMENT: LESSONS FROM THE HEARS RCT

**DOI:** 10.1093/geroni/igae098.0431

**Published:** 2024-12-31

**Authors:** Carrie Nieman, Joshua Betz, Jonathan Suen, Jami Trumbo, Hae-Ra Han, Nicole Marrone, Frank Lin, Esther Oh

**Affiliations:** Johns Hopkins University School of Medicine, Baltimore, Maryland, United States; Cochlear Center for Hearing & Public Health, Baltimore, Maryland, United States; Johns Hopkins University, Baltimore, Maryland, United States; Johns Hopkins University, Baltimore, Maryland, United States; Johns Hopkins University, Baltimore, Maryland, United States; University of Arizona, Tucson, Arizona, United States; Johns Hopkins University, Baltimore, Maryland, United States; Johns Hopkins University, Baltimore, Maryland, United States

## Abstract

Hearing loss is highly prevalent and associated with adverse health outcomes but undertreated among individuals with cognitive impairment, particularly African Americans. The incorporation of community health worker (CHW)-partnered models may increase access and reduce disparities. The HEARS intervention is a hearing care intervention delivered by CHWs that provides a low-cost amplification device. This pre-specified subgroup analysis based within the HEARS randomized controlled trial (RCT) aims to assess whether cognitive impairment modified the effect of the HEARS intervention among community-dwelling older adults. The analysis was stratified by cognitive status using the total Montreal Cognitive Assessment (MoCA) score (□25: cognitive impairment; post hoc sensitivity analysis using □22). Among 149 randomized participants with MoCA data, 100 individuals were cognitively impaired (Mean adjusted MoCA: 21(SD 3.5; 52% African American; 70% low-income). At 3-months post-intervention, communication function significantly improved among individuals with cognitive impairment compared with control, with an estimated average treatment effect of -13.92 HHIE-S change (95% CI:-16.84,-10.86), comparable to those without cognitive impairment (-11.47; 95% CI:-18.04,-4.17). Post hoc sensitivity analysis using a □22 MoCA cut-off for cognitive impairment yielded similar findings. Among individuals with cognitive impairment, the HEARS intervention, compared with waitlist control, significantly improved communication function. The improvements were comparable to participants without cognitive impairment and similar in magnitude to improvements documented for older adults who received conventional hearing care. To the authors’ knowledge, this trial was the largest trial to date of a hearing care intervention in the U.S. of African American older adults and low-income older adults with cognitive impairment.

